# Hostile Attribution Bias and Anger Rumination Sequentially Mediate the Association Between Trait Anger and Reactive Aggression

**DOI:** 10.3389/fpsyg.2021.778695

**Published:** 2022-01-12

**Authors:** Fangying Quan, Lu Wang, Xinyu Gong, Xiaofang Lei, Binqi Liang, Shuyue Zhang

**Affiliations:** ^1^Department of Psychology, Faculty of Education, Guangxi Normal University, Guilin, China; ^2^Research Center for the Development of Guangxi Ethnic Education, Guilin, China; ^3^Guangxi University and College Key Laboratory of Cognitive Neuroscience and Applied Psychology, Guilin, China; ^4^Faculty of Psychology, Beijing Normal University, Beijing, China

**Keywords:** trait anger, reactive aggression, hostile attribution bias, anger rumination, mediation

## Abstract

Reactive aggression is a type of aggression that has severe consequences in individual’s psychosocial development and social stability. Trait anger is a risk personality factor for reactive aggression. However, the mediating mechanism of this relationship has not been sufficiently analyzed. We proposed that hostile attribution bias and anger rumination may be cognitive factors that play mediating roles in the relationship between trait anger and reactive aggression. To test this hypothesis, a sample of 600 undergraduates (51.67% females, *M*_age_ = 20.51, SD = 1.11) participated in this study. Findings showed that hostile attribution bias, anger rumination sequentially mediated the association between trait anger and reactive aggression. These results highlight the importance of anger rumination and hostile attribution bias to explain the link between trait anger and reactive aggression in undergraduates. The findings of the present study also provide valuable information about the role of negative cognitive activities (e.g., hostile attribution, ruminate in anger emotion) in high trait anger individual may trigger reactive aggression. The limitations of the study are discussed, along with suggestions for future research.

## Introduction

The prevalence of aggression is a significant social concern, having destructive effects not only for the victims, but also for our society. Aggression can cause different consequences such as vandalism, robbery, assault, rape, and so on (Zhou et al., 2017). According to the motivation, aggression is often divided into two distinct subtypes: reactive and proactive (Dodge and Coie, 1987; Li and Xia, 2021). Among them, reactive aggression is a defensive, impulsive response to a perceived threat or provocation, referring to an emotionally charged (e.g., highly emotionally aroused, anxious, and angry) aggression that has also been described as impulsive, hot-blooded, or affective (Dodge and Coie, 1987; Euler et al., 2017). In the same way, it is an aggressive subtype with disinhibition, and occurring in response to provocation or threat by others (Dodge and Coie, 1987; Liu and Liu, 2021). For example, individual who gets mad after being accused, scold or provoke by a peer and then pushing the peer demonstrating reactively physically aggressive behavior. This kind of aggression is related to anxiety, depression, and peer rejection (Bondü and Richter, 2016). Given the great harm and serious consequences that results from reactive aggression, it is necessary to understand the development of reactive aggression comprehensively and deeply. So, it is of theoretical and practical value to explore and understand the influencing factors and the mechanism of reactive aggression. The risk of developing reactive aggression may increase underneath some certain personality features. Trait anger referring to the stable individual differences in the frequency, intensity, and duration of state anger episodes (Deffenbacher et al., 1996; Wilkowski and Robinson, 2008, 2010), has been well documented as a personality variable that most strongly linked to aggression. Regarding the literature, underlying mechanism responsible for the relationship between trait anger and reactive aggression remains unclear. To carry out targeted prevention and intervention of reactive aggression, we try to explore the relationship between trait anger and reactive aggression and unveil its mediating mechanism in this study.

### Trait Anger and Reactive Aggression

High level of trait anger is related to adverse consequences such as increased aggression in our daily life, in particular reactive aggression. The General Aggression Model (GAM) postulates that aggressive behavior occurs as a result of the interplay between individual (e.g., personality traits) and situational (e.g., presence of a provocation, or an aggressive cue) factors, and aggression-related personality factors also can affect individuals’ aggression (Anderson and Bushman, 2002). As a personality constructs, the tendency that anger toward a perceived threat is one of the crucial input variables that leads individuals to attack others. Integrative Cognitive Model (ICM; Wilkowski and Robinson, 2010) of trait anger and reactive aggression also proposes that trait anger is the susceptibility factor of reactive aggression. Empirical studies have confirmed that trait anger is an important contributor to aggression in provoking situations (Hubbard et al., 2010; Robinson and Wilkowski, 2010). For example, cross-sectional study and a longitudinal study were showed a significant correlation between trait anger and reactive aggression (Bondü and Richter, 2016; Li and Xia, 2021). Thus, we propose our first hypothesis: trait anger can predict reactive aggression.

According to the ICM (Wilkowski and Robinson, 2010), hostile attribution bias and ruminative attention are important cognitive processes for understanding individual differences in trait anger and reactive aggression. Specifically, individual who high on trait anger are automatically more likely to interpret ambiguous situations as hostile, and this in turn lead to more frequent elicitation of state anger and reactive aggression. In addition, hostile attribution bias also can take on the automatic processes of ruminative attention (such as anger rumination), which in turn can amplify state anger and reactive aggression (Wilkowski and Robinson, 2010). Although valuable work has been done on the relationship between trait anger and reactive aggression. That is to say, empirical studies on these two psychological mechanisms (hostile attribution bias and anger rumination) in trait anger and reactive aggression are still limited. Therefore, our main purpose is to explore the mediating role of hostile attribution bias and anger rumination in the relationship between trait anger and reactive aggression.

### Hostile Attribution Bias as a Mediator Between Trait Anger and Reactive Aggression

The tendency to perceive as, or attribute to, hostile intent the ambiguous action of others has been termed hostile attribution bias (Kokkinos et al., 2017) or hostile attribution style (Dodge, 2006). We hypothesized that one of the mediators among the relationship between trait anger and reactive aggression is hostile attribution bias. The reasons are followed. First, trait anger may trigger hostile attribution bias. Previous studies (e.g., Veenstra et al., 2017; Li and Xia, 2021) supported the idea that trait anger is one of the predictors of hostile attribution bias. Fitzgibbons (1986) found that anger caused by offensive life events can promote the formation of a psychological defense mechanism, which reduce the individual’s compassion and empathy for the offender and promote the generation of hostile thinking toward others. Individuals who with a high level of trait anger have negative perceived bias when in face of threat-related information (Smith and Waterman, 2003), and more sensitive to hostile social cues (Wilkowski and Robinson, 2007; Li and Xia, 2021). Trait anger also related to faster reading of sentences that described angry reactions to ambiguously anger-provoking situations (Wingrove and Bond, 2005). Thus, we inferred that. Trait anger makes individuals easier to interpret others’ intention as hostile in ambiguous situations just because they have negative perceived bias and pay more attention to hostile cues. Second, hostile attribution bias can predict reactive aggression. According to the Social Information Processing (SIP) model, individuals who misinterpret the behavior of others as intentionally harmful to themselves would be more likely to react aggressively (Dodge and Coie, 1987; Crick and Dodge, 1994). Previous research on children (Hubbard et al., 2010; Dodge et al., 2015), adolescents (Kokkinos et al., 2017), and adults (Bondü and Richter, 2016; Li and Xia, 2021) have demonstrated the robust relationship between hostile attribution bias and reactive aggression. Specifically, Dodge et al. (2015) showed that, among 12 diverse ecological-context groups in nine countries worldwide, children who attributed hostile intent to others in response to provocation reflect a key psychological process that accounts for individual differences in reactive aggression. Hostile attribution bias could predict reactive aggression, but not proactive aggression across time when controlling for gender and age (Quan and Xia, 2019). Studies have also shown that trait anger and hostile attribution bias can sequentially mediate the effects of childhood punishment experience on undergraduate student authoritarianism (Milburn et al., 2014). Consequently, we put forward our second hypothesis: hostile attribution bias plays a mediating role in the relationship between trait anger and reactive aggression.

### Anger Rumination as Another Mediator Between Trait Anger and Reactive Aggression

In addition to the mediating role of hostile attribution bias, anger rumination is also an important psychological mechanism for trait anger predicting reactive aggression. Rumination attention can be defined as focusing one’s attention on negative information, including anger information (Wilkowski and Robinson, 2008). Anger rumination involves thinking continuously about angry moods, the consequences and causes of anger events, and can’t stop thinking about how to revenge (Sukhodolsky et al., 2001; Denson, 2013; Quan et al., 2019). According to the ICM, anger rumination can be thought as a selective attention process (Wilkowski and Robinson, 2008, 2010) and it is a vital component of ruminative attention. As a result, anger rumination may be another cognitive factor that related to trait anger and reactive aggression. The following is the opinions and research evidence. First, trait anger can contribute to anger rumination because it is characterized by a greater inclination toward ruminative attention to hostile thoughts (Wilkowski and Robinson, 2010). The findings of a number of correlational studies suggest that trait anger is positively correlated with anger rumination (Peters et al., 2014; Borders and Lu, 2017). Specifically, anger rumination may, at least partially, account for the predictor role of trait-level anger on borderline personality features (Baer and Sauer, 2011). Second, systems model of anger rumination proposes that it takes more effort to regulate one’s internal state when experiencing angry, and this effort consumes cognitive resources. The loss of cognitive resources can trigger individual aggression (Denson, 2013). Because reactive aggression is one of the functions of aggression, anger rumination can perhaps exacerbate tendencies toward reactive aggression. One study described an undergraduate student whose anger rumination was associated with reactive aggression, even after controlling for proactive aggression (White and Turner, 2014). Moreover, in the experimental studies, anger rumination can increase aggressive behavior (Bushman, 2002; Bushman et al., 2005). This may be one of the reasons why anger rumination tends to trigger reactive aggression. To our knowledge, only few studies have examined the association among anger, anger rumination, and aggression. The result showed that anger rumination mediates the relationship between trait driving anger and aggressive driving behaviors (Suhr and Nesbit, 2013) and aggression (Hou et al., 2017; Wang X. et al., 2018). However, whether anger rumination plays a mediating role in the relationship between trait anger and reactive aggression in college students still needs exploring. Therefore, we propose our third hypothesis: anger rumination is another mediator in the relationship between trait anger and reactive aggression.

### Hostile Attribution Bias and Anger Rumination as Serial Mediators Between Trait Anger and Reactive Aggression

According to the ICM, hostile attribution bias and anger rumination could not only mediate the relationship between trait anger and reactive aggression, but also play a chain role in this relationship. Some studies also showed that hostile attribution bias can affect aggression through anger rumination (Quan et al., 2019; Li and Xia, 2020). Hostile attribution bias showed a significant predictive effect on anger rumination 6 months later (Wang et al., 2019). Furthermore, hostile attribution involves the elicitation of angry feelings (Wilkowski and Robinson, 2008, 2010). The key characteristics of anger rumination can be accompanied by angry feelings (Sukhodolsky et al., 2001; Denson, 2013). Maybe hostile attribution bias and anger rumination both mediate the link between trait anger and reactive aggression. Thus, our last hypothesis is that hostile attribution bias and anger rumination as serial mediators between trait anger and reactive aggression.

### Present Research

In summary, a substantial body of empirical studies suggested that trait anger is the risk factor of reactive aggression; however, the underlying mechanism of the relationship remains unclear. In the current study we aimed to extend previous research. We aimed to examine whether trait anger increases the likelihood or frequency of reactive aggression in daily life by increasing the individuals’ hostile attribution and anger rumination level. To test the mediating roles of hostile attribution bias as well as anger rumination, this study mainly aims to investigate the relationship between trait anger and reactive aggression among college students. Specifically, there are four hypotheses will be examined: (1) trait anger positively related with reactive aggression; (2) hostile attribution bias is a mediator in the relationship between trait anger and reactive aggression; (3) anger rumination is another mediator in the relationship between trait anger and reactive aggression; (4) hostile attribution bias and anger rumination play serial mediating role in the relationship between trait anger and reactive aggression.

## Materials and Methods

### Participants

A total of 600 verified undergraduates participated in the current study. Among them, 51.67% were female and 48.33% were male (*M*_age_ = 20.51, SD = 1.11). They were recruited from Southwest University, Guangxi Normal University and the Nanyang Institute of Technology in China. After being provided with a complete description of the study, all the participants gave written informed consent. This study was approved by the Academic Ethics Committee of Faculty of Education, Guangxi Normal University.

### Materials

#### Trait Anger

Trait Anger Subscale (TAS) of the State-Trait Anger Expression Inventory-2 (STAXI-2; Spielberger, 1999) was used to measure trait anger; participants were asked to rate 10 items (e.g., I get angry when slowed down) on a 4-point Likert scale, from 1 “completely disagree” to 4 “completely agree.” STAXI-2 has been translated into many languages, including Chinese version (Maxwell et al., 2009; Khodayarifard et al., 2013; Wang Y. et al., 2018). The Chinese version of TAS has good validity and reliability, and was suitable for measuring the Chinese college students (Maxwell et al., 2009; Wang Y. et al., 2018; Li and Xia, 2020, 2021). Higher scores on the TAS represent a greater tendency to become angry frequently and intensely. The Cronbach’s alpha coefficient of the total TAS in this sample is 0.86.

#### Hostile Attribution Bias

We measured hostile attribution bias Social Information Processing–Attribution Bias Questionnaire (SIP–ABQ; Coccaro et al., 2009). The measure included eight short vignettes, each describing an ambiguous social situation with a negative outcome that may have been caused intentionally or unintentionally (e.g., early one morning, you go to a busy local coffee shop to get a cup of coffee. While you are waiting, someone you see at the coffee shop regularly, but do not know personally, cuts in the line in front of you). Participants rated the likelihood of two explanations for the outcome, signaling hostile intent per scenario as 0 (not at all likely) to 3 (very likely). For the purpose of this study, the average score across the 16 items was taken as the indicator of hostile attribution bias (Coccaro et al., 2009). The Cronbach’s alpha coefficient for hostile attribution bias in this sample is 0.89.

#### Anger Rumination

Anger Rumination Scale (ARS; Sukhodolsky et al., 2001) was developed to assess the tendency to think about current anger-provoking situations, and the ruminative processes the anger provoked in the past, including angry memories (e.g., I feel angry about certain things in my life), thoughts of revenge (e.g., I have long-living fantasies of revenge after the conflict is over), angry after thoughts (e.g., after an argument is over, I keep fighting with this person in my imagination), and an understanding of the causes (e.g., I think about the reasons people treat me badly). Participants rated the 19-item ARS from 1 (not at all) to 4 (almost always). ARS has a good reliability among Chinese college students (Hou et al., 2017). All 19 items were added together to assess anger rumination, and the ARS values demonstrated good reliability (α = 0.93) in the sample.

#### Reactive Aggression

Reactive Aggression Subscale (RAS) of the Reactive–Proactive Aggression Questionnaire (RPQ; Raine et al., 2006) was used to measure reactive aggression. The 11-item (e.g., reacted angrily when provoked by others) RAS asked participants to rate each question, in terms of frequency, on a 3-point Likert scale. There is a good reliability of RAS among Chinese college students (Quan and Xia, 2019). In the present study, Cronbach’s coefficient alpha of the RAS is 0.81.

### Procedure

This survey was conducted by trained research assistants, after obtaining informed consent, the participants were asked to complete the series of questionnaires in a classroom. Participants were told that the survey was not a test, that there were no right or wrong answers, then they were instructed to answer the questions as honestly as possible. In addition, we told the students that the survey was anonymous and that the information they filled would only be used for scientific research and would not be shared with others. To reduce the common method variance, two versions of the A and B questionnaires were used to balance the order of measurement of different variables (Podsakoff et al., 2003; Quan et al., 2021). That is, the order of the TAS, SIP–ABQ, ARS, and RPQ-RA was counterbalanced. Half of the participants completed the version of questionnaires in the order of TAS, SIP–ABQ, ARS, and RPQ-RA, while in version B the other half of the participants completed the questionnaires in RPQ-RA, ARS, SIP–ABQ, and TAS.

### Statistical Analyses

Prior to performing a mediation analysis, common-method bias tests, descriptive statistics, and correlations analyses were performed using SPSS25.0. Firstly, correlations among all study variables, including age and gender were estimated to determine univariate associations. Structural equation modeling with latent variables *via* Mplus7.0 was used to examine the hypothesized mediating model. To control for measurement error, we used the robust maximum likelihood estimation to deal with non-normal data and missing values. Relational analysis followed the two-step procedure: the measurement model was first analyzed to assess the extent to which each latent variable was represented by its indicators. If the measurement model was accepted, then the structural model was tested (Anderson and Gerbing, 1988). The excellent fit of the model was also assessed in the both measurement and structural models, the criteria as following: χ^2^/df ratio < 3, RMSEA < 0.06, SRMR < 0.08, both CFI and TLI > 0.95 (Hu and Bentler, 1999). To test the entire hypothesis mentioned above, the structural model was conducted using Mplus7.0. The independent variable was trait anger, the dependent variable was reactive aggression, hostile attribution bias and anger rumination both were mediating variables, age, and gender were included as covariance. Thousand samples of bias-corrected bootstrapping were sampled. If the 95% of confidence intervals did not include zero, that can mean the indirect effects of hostile attribution bias and anger rumination were significant.

## Results

### Common-Method Bias Test

Harman single-factor test (Podsakoff et al., 2003) was used to test the common method bias. The results showed that there were 11 factors with an Eigen root greater than 1, among which the first common factor explained 24.34% of the variance, which was lower than the 40% threshold value. Thus, the data in this study had no serious common method bias problem.

### Descriptive Statistics and Correlation Analysis

Descriptive statistics and correlation analysis were conducted using SPSS25.0. Previous studies have shown that age and gender may related to aggression or anger rumination (Zsila et al., 2019; Toro-Tobar et al., 2020), therefore, age and gender were included in the correlation analysis. As predicted, analyses revealed significant relationships among all the studied variables (see Table 1). Trait anger was significantly correlated with hostile attribution bias, anger rumination and reactive aggression. Hostile attribution bias was significantly correlated with anger rumination and reactive aggression. Anger rumination was significantly correlated with reactive aggression. Gender was only significantly correlated with anger rumination, and age was not related to all the other variables. In order to reduce the potential influence of gender and age on the relationship between the study variables, these two variables will be included as control variables in the following structural equation model.

**TABLE 1:** The results of descriptive statistics and correlation analysis (*N* = 600).

Variable	*M*	SD	Age	Gender	TA	HAB	AR
Age	20.51	1.11	–				
Gender	–	–	–	–			
TA	1.61	0.45	0.007	0.02	–		
HAB	1.11	0.42	0.02	0.01	0.34***	–	
AR	2.81	1.25	0.02	0.16***	0.49***	0.37***	–
RA	0.67	0.32	–0.03	0.001	0.55***	0.37***	0.51***

*TA, trait anger; HAB, hostile attribution bias; AR, anger rumination; RA, reactive aggression.*

***p < 0.001.

### Measurement Model

Confirmatory factor analysis was used to test the measurement model. Item parcels can improve the stability of the latent variables (Little et al., 2002) and reduce the sampling error sources (Little et al., 2013). In order to improve the psychometric properties of the variables and control for inflated measurement errors, all variables were treated as latent variables, and were divided into four parcels (composed of two to six items each). The building parcels technique for trait anger, hostile attribution bias and reactive aggression is the balancing technique. The multidimensional anger rumination was constructed on parcels in the internal-consistency approach (Little et al., 2013); the advantage of the internal-consistency approach is that the multidimensional nature of the construct can be kept explicit. The fit indices of the measurement model were good: χ^2^/df ratio = 1.74, *p* < 0.001, RMSEA = 0.04 (90% CIs [0.03, 0.04]), CFI = 0.98, TLI = 0.98, SRMR = 0.03. All factor that loadings above 0.68 for the indicators of the latent factors were significant (*p* < 0.001), which suggest that the latent variables were well represented by their respective indicators.

### Structural Model

**FIGURE 1:**
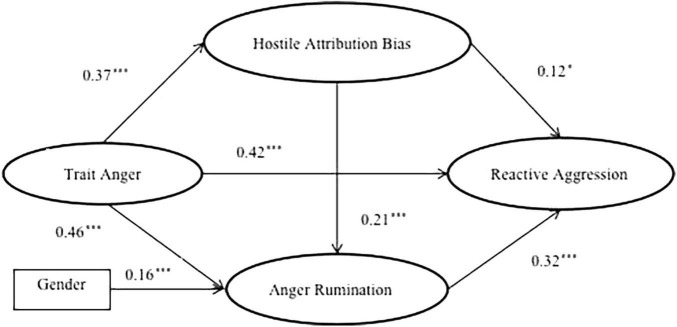
The final model of chain mediation model. Only the significant standardized coefficients were showed in this final model. *p < 0.05, ^***^p < 0.001.

We tested the proposed model, in which trait anger is correlated to reactive aggression through the mediation role of hostile attribution bias as well as anger rumination for controlling for gender and age. The results indicated that the hypothesized mediation model has a good fit to the data: χ^2^/df ratio = 2.49, *p* < 0.001, RMSEA = 0.05 (90% CIs [0.04, 0.06]), CFI = 0.97, TLI = 0.96, SRMR = 0.03. As presented in Figure 1, the pathway parameter estimates show that all the path coefficients were significant in the proposed directions. Specifically, after controlling for gender and age, the total effect of trait anger on reactive aggression was significant (β = 0.64, *p* < 0.001). Direct effects of trait anger on reactive aggression (β = 0.42 *p* < 0.001), hostile attribution bias (β = 0.37, *p* < 0.001) and anger rumination (β = 0.46, *p* < 0.001) were significant. Direct effects of hostile attribution bias on reactive aggression (β = 0.12, *p* < 0.05) and anger rumination (β = 0.21, *p* < 0.001) were significant. Direct effects of anger rumination on reactive aggression (β = 0.32, *p* < 0.001) also was significant.

Meanwhile, the bootstrapping procedure in Mplus7.0 was used to test the significance of the mediating effects, bias-corrected bootstrapping with 1,000 samples was examined, and the significant indirect effects are indicated by 95% CIs that do not include zero. Indirect effects and their associated 95% confidence intervals were displayed in Table 2. The results of the structural model are shown. The total indirect effects were significant (β = 0.22, 95% CI [0.16, 0.28]). The indirect effect value produced by the way of trait anger → hostile attribution bias → reactive aggression is 0.05, 95% CI: 0.01–0.09; the indirect effect of trait anger → anger rumination → reactive aggression was 0.15, 95% CI: 0.10–0.20. The indirect effect of trait anger → hostility attribution bias → anger rumination → reactive aggression was 0.03, 95% CI: 0.01–0.04. These results supported the three mediating models’ hypothesis, which implied that hostile attribution bias and anger rumination both mediated the trait anger-reactive aggression linkage, respectively, and play serially mediating role in this relationship.

**TABLE 2:** Standardized indirect effects and 95% confidence intervals.

Mediate pathways	Point estimate	95% CIs		Proportion of indirect effects (%)
		Lower	Upper	
TA–HAB–RA	0.05	0.01	0.09	7.20
TA–AR–RA	0.15	0.10	0.20	22.80
TA–HAB–AR–RA	0.03	0.01	0.04	3.90
Total indirect effect	0.22	0.16	0.28	34.10

*HAB, hostile attribution bias; AR, anger rumination; TA, trait anger; RA, reactive aggression.*

## Discussion

It is vital to note that previous studies primarily focused on the direct relation between trait anger and reactive aggression. As mentioning above, ICM mainly explored the psychological mechanism of trait anger predicting reactive aggression. However, empirical evidence of this model is still very limited. Therefore, based on ICM and questionnaire survey, this study examined the relationship between trait anger, hostile attribution bias, anger rumination and reactive aggression by establishing structural equation model. The results support our four hypotheses: (1) trait anger was positively correlated with reactive aggression; (2) hostile attribution bias as mediator between trait anger and reactive aggression; (3) anger rumination as mediator between trait anger and reactive aggression; and (4) hostile attribution bias and anger rumination as serial mediators between trait anger and reactive aggression. Therefore, this study adds evidence to support the mechanism of the relationship between trait anger and reactive aggression according to ICM.

Regarding the first goal, the results of this study support the hypothesis that trait anger is an important personality factor that predicts reactive aggression. This finding enriches prior studies (e.g., Ann et al., 2006; Wilkowski and Robinson, 2010; Li and Xia, 2021). Meta-analytic review suggested that when provoked, individuals who were high-ranking in trait anger showed reliably greater levels of aggression than those low-ranking in trait anger (Ann et al., 2006). Laboratory-based studies (Bushman et al., 2001; Leki and Wilkowski, 2017) also revealed that individuals who ranked high in trait anger behaved more aggressively under provocation rather than under unprovoked conditions. The experience of anger in pushing an individual to adopt defensive aggression to relieve unperceived threat (Gagnon and Rochat, 2017), which directly promotes the individual’s reactive aggression. Our finding implies that trait anger is an important personality factor leading to reactive aggression.

### Mediating Effect of Hostile Attribution Bias

Consistent with our second hypothesis, hostile attribution bias play a mediating role in the relationship between trait anger and reactive aggression. Regard of the result that trait anger was significantly associated with hostile attribution bias, several studies also supported this link (Orue et al., 2019; Li and Xia, 2021). For instance, hostile attribution has been found among adults who are prone to anger (Epps and Kendall, 1995; Dill et al., 1997; Milburn et al., 2014; Orue et al., 2019). Trait anger can predict hostile attribution among adolescents 1 year later (Orue et al., 2019) and among undergraduate students 6 months later (Li and Xia, 2021). Besides the theoretical viewpoint of ICM and General Affective Aggression Model (Anderson et al., 1995), people with aggressive personalities (e.g., trait anger) tend to view the world through blood-red-tinted glasses, in turn, perceiving and understanding social events more hostile than people with less aggressive personalities. Furthermore, trait anger also can make individuals tend to process hostile events (Veenstra et al., 2018) in ambiguous situations. Trait anger may cause individuals tend to interpret perceived provocation from a negative perspective, interpreting the intention of others’ action as hostile (Li and Xia, 2021). As presented in Figure 1, people who interpret ambiguous situations as intentionally hostile are more likely to aggress against a provocateur. This result supported the SIP model (Crick and Dodge, 1994) which explicates how reactive aggression is predicted by hostile attribution bias. SIP model outlines that if an individual misinterprets hostile intent to observed behaviors, she/he may select aggressive behaviors as appropriate responses to perceived provocation (Dill et al., 1997). Research conducted on adults has consistently indicated a robust association between hostile attribution bias and reactive aggression (Bailey and Ostrov, 2008; Bondü and Richter, 2016; Gagnon and Rochat, 2017). For example, hostile attribution bias has been more strongly linked to relational and physical reactive aggression (Bailey and Ostrov, 2008).

In summary, the mediating effect of hostile attribution supports the viewpoint among ICM that people who show intense anger tend to attribute hostility to others’ behavior, which increases reactive aggression (Wilkowski and Robinson, 2010). That is, evidence from theories and studies suggest that individual ranking high in trait anger are prone to adopt hostile interpretations to relieve the perceived threat, and then responding to provocation with more defensive and aggressive behaviors.

### Mediating Effect of Anger Rumination

One of our results supported the third hypothesis: anger rumination can independently mediate the relationship between trait anger and reactive aggression. Similar to the result of this study, existing studies (Suhr and Nesbit, 2013; Li and Xia, 2020) have shown that trait anger was significantly correlated with anger rumination. For instance, individuals who rank high in trait anger are less likely to be able to control anger rumination (Suhr and Nesbit, 2013). Trait anger can predict undergraduate students’ anger rumination across 6 months later (Li and Xia, 2020). There are several interpretations of this association. First, recurrent negative thinking is an underlying characteristic of high trait anger (Owen, 2011), and anger rumination is a cognitive process that begins following an anger-induced event (Denson, 2013). Thus, we speculate that anger rumination is a consequence variable of trait anger. Second, ICM posits that individuals ranking high in trait anger should display selective attention processes, favoring hostile information, which in turn may facilitate rumination in relation to them (Wilkowski and Robinson, 2008). This cognitive view of rumination has been supported by relevant study (Smith and Waterman, 2005), which have found that individuals ranking higher in trait anger were faster to respond to hostile, relative to non-hostile stimuli. The results indicated that individuals with greater tendencies toward anger may easily migrate toward anger-related information and moods. Besides, the results also showed that gender has a significant effect on anger rumination, which means that after controlling the potential effect of gender, the predictive effect of trait anger) on anger rumination is still significant. Figure 1 showed that anger rumination is a risk factor for reactive aggression. This result was consistent with the previous study finding that anger rumination can predict reactive aggression after a 6 month period (Wang et al., 2020), it also supported and expanded our understanding of the multiple systems model (Denson, 2013). According to the multiple systems model, anger rumination following a provocation increases impulsive aggression and decreases the likelihood of refraining from aggression, which can be due to the failure of self-control (Denson et al., 2011). Thus, anger rumination may be a common predictor for reactive aggression.

Therefore, our results indicate that anger rumination is one of the crucial mental mechanisms underlying the role of trait anger acts on reactive aggression. Moreover, the mediating model supports a theoretical view which described trait anger might influence an individual’s propensity to aggression through increasing their attention to provocative events (Anderson and Bushman, 2002; Wang Y. et al., 2018). This mediating model also can be explained by self-regulatory capacities for resolving anger. Specifically, individuals who have high-ranking trait anger usually have a deficiency of self-regulatory capacities for resolving anger (Aricak and Ozbay, 2016) due to their focus on anger feelings and revenge thoughts. Individuals ruminated in anger are usually unable to effectively manage their anger, which is more likely to lead to reactive aggressive behavior.

### Mediating Role of Anger Rumination on the Association Between Hostile Attribution Bias and Reactive Aggression

Figure 1 also showed that hostile attribution bias can predict anger rumination. It means that hostile attribution bias and anger rumination can sequentially mediate the association between trait anger and reactive aggression. This result supported the fourth hypothesis, and consistent with previous studies (Quan et al., 2019; Wang et al., 2019). People make hostile attributions based on hostile schemas (Dodge, 2006). Individuals who make hostile attributions may find it easier to memorize and immerse in anger-inducing events, so, hostile attribution bias may trigger anger rumination. Furthermore, hostile attribution bias involves a scribing the intent of others as hostile. Anger rumination also results from analyzing the causes, consequences and meaning of anger information (Sukhodolsky et al., 2001). The ambiguity of intentions would induce individuals to ruminate on the causes of angry events, even after the events have ended. In summary, individuals ranking high in trait anger automatically interpret ambiguous situations as having hostile intent. This attribution bias is likely to cause individuals more easily proceed with ruminative processes, ultimately increasing the level of reactive aggression.

### Limitations and Future Directions

Several important limitations should be considered when interpreting these results. Firstly, using the method of self-report based on cross-sectional study cannot determine the causal relationship between the variables, and whether the findings would translate into actual aggressive acts. Furthermore, it is uncertain that whether the correlations amongst trait anger, hostile attribution bias, anger rumination and reactive aggression were mainly due to the positive–negative response set. Therefore, in the future studies, longitudinal, observational, or experimental studies should be adopted. Meanwhile, using other assessment methods (e.g., parental rating, teacher rating, peer nomination) to measure the study variables is also a way to avoid the positive–negative response set. Secondly, our sample was only collected from college student; the generalizability may be somewhat limited. Future work should attempt to replicate our mediation findings on other groups, such as older workers, community individuals and violent offenders. Thirdly, it is showed that the average score of the variables (such as trait anger, hostile attribution bias, and reactive aggression) is low. This may lead the findings cannot generalize to the general population or the people who high-level trait anger, hostility attribution bias and reactive aggression. This suggests that these findings are only a preliminary, whether it is universal still needs to be tested in future studies. Another limitation is that we only discuss the mediating mechanism between the relationship of trait anger and reactive aggression. Given that hostile attribute bias and anger rumination can both amplify state anger, and effortful control may moderate the relationship between trait anger and reactive aggression (Wilkowski and Robinson, 2010). Future researches can explore the other mental mechanisms, such as state anger and effortful control, underlying the relation between trait anger and reactive aggression.

### Theoretical and Practical Contributions

Notwithstanding these limitations, this study provides some theoretical and practical contributions. From a theoretical perspective, our study provides an empirical framework for researchers – through testing the cognitive mechanism of trait anger on reactive aggression – and the findings can shed light on the mediating mechanisms between trait anger and reactive aggression. Exploring the relationship of these four variables, not only develop the theory of the relationship between personality (e.g., trait anger) and aggression, but also provide a theoretical framework for in-depth understanding of the internal mechanism of personality susceptibility factors leading to aggressive behavior (Li and Xia, 2021). From a practical perspective, these findings are essential for a better understanding of the etiology of reactive aggression among college students who have high-ranking trait anger. These results may also help in developing effective intervention programs to prevent and reduce reactive aggression among individuals who have high-ranking trait anger. For instance, a hostile attribution bias modification paradigm (AlMoghrabi et al., 2018) may be effective in reducing hostile attribution bias, and developing mindfulness training to reduce anger rumination (Wang Y. et al., 2018) would also help to decrease reactive aggression.

## Conclusion

This study preliminarily showed that reactive aggression is positively correlated with trait anger, hostile attribution bias and anger rumination. In addition, this study also found two mediating variables (hostile attribution bias and anger rumination) in the relationship between trait anger and reactive aggression. Previous ICM are preliminarily supported and developed by these findings.

## Data Availability Statement

The original contributions presented in the study are included in the article/supplementary material, further inquiries can be directed to the corresponding author.

## Ethics Statement

This manuscript has not been published or presented elsewhere in part or in entirety and is not under consideration by another journal. The study was carried out in accordance with the recommendations of the Academic Ethics Committee of Faculty of Education, Guangxi Normal University with written informed consent from all participants.

## Author Contributions

FQ conception or design, collection, analysis or interpretation of data, and writing – review and editing. LW, XG, XL, and BL revising it critically for important intellectual content. SZ revising critically for theoretical framework, important intellectual content, and language expression of the manuscript. All authors approved the final version of the manuscript for submission.

## Conflict of Interest

The authors declare that the research was conducted in the absence of any commercial or financial relationships that could be construed as a potential conflict of interest.

## Publisher’s Note

All claims expressed in this article are solely those of the authors and do not necessarily represent those of their affiliated organizations, or those of the publisher, the editors and the reviewers. Any product that may be evaluated in this article, or claim that may be made by its manufacturer, is not guaranteed or endorsed by the publisher.
